# The Protective Effect of Vitamin D Against Necroptosis in Preeclampsia

**DOI:** 10.1155/jp/4106792

**Published:** 2026-03-31

**Authors:** Atikah Sayogo Putri, Rima Irwinda, Noroyono Wibowo

**Affiliations:** ^1^ Department of Obstetrics and Gynecology, Universitas Indonesia, Cipto Mangunkusumo Hospital, Jakarta, Indonesia, rscm.co.id; ^2^ Division of Maternal–Fetal Medicine, Department of Obstetrics and Gynecology, Universitas Indonesia, Cipto Mangunkusumo Hospital, Jakarta, Indonesia, rscm.co.id

**Keywords:** cell death, inflammation, necroptosis, preeclampsia, pregnancy, vitamin D

## Abstract

**Objective:**

Preeclampsia is correlated with an inflammatory condition. Necroptosis is a programmed cell death with an inflammatory state. Vitamin D has anti‐inflammatory properties; however, there has been no study linking vitamin D and necroptosis in preeclampsia. This study is aimed at evaluating vitamin D status and necroptosis activity in preeclampsia.

**Methods:**

A cross‐sectional study was conducted in Jakarta during 2021–2023. Subjects were grouped into normal and preeclampsia. Following delivery, venous blood and placental samples were taken. Serum and placental 25(OH)D assays were performed by LC‐MS/MS. Immunohistochemistry was performed to measure necrosomes RIPK1, RIPK3, and MLKL in trophoblast and endothelial.

**Results:**

A total of 60 subjects participated (31 normal and 29 preeclampsia). The preeclampsia group had lower gestational age (35 vs. 38 weeks), lower birth weight (3080.33 ± 454.62 g vs. 2283.27 ± 833.63 g), lower placental weight (580.40 ± 129.36 g vs. 453.06 ± 173.65 g), lower placental 25(OH)D (15.00 [3.50–58.00] vs. 26.50 [5.00–153.00] ng/mL, *p* = 0.014), and higher trophoblast RIPK3 (93.88 [23.94] vs. 76.20 [20.59], *p* = 0.003). A mild to moderate negative correlation between placental 25(OH)D and trophoblast RIPK3 (−0.352, *p* = 0.003), endothelial RIPK3 (*r* = −0.244, *p* = 0.03), and trophoblast MLKL (*r* = −0.296, *r* − 0.011) were observed.

**Conclusion:**

Lower placental 25(OH)D concentration is associated with an increased placental necroptosis activity in preeclampsia.

## 1. Introduction

Maternal mortality rate remains a significant issue in Indonesia, reaching 305 per 100,000 live births [1]. Hypertension in pregnancy accounts for approximately 19% of maternal deaths in Indonesia [2]. Preeclampsia is a major cause of maternal mortality and morbidity, preterm birth, perinatal death, and intrauterine growth restriction [3].

Preeclampsia is a multisystem disorder with a multifactorial etiology. There have been numerous hypotheses of its pathophysiology; however, it is agreed that the clinical syndrome begins with abnormal placentation with subsequent release of antiangiogenic markers. This event results in endothelial dysfunction, vasoconstriction, and immune dysregulation, which can negatively impact various maternal organ systems as well as the fetus [4].

Microscopically, the placenta in preeclampsia shows more syncytial knots, hypertrophy of the spiral artery, and necrotic villi. These findings have led to a deeper study particularly about cell death in preeclampsia. There are several mechanisms of cell death, among which are necrosis, apoptosis, and necroptosis. Although increased placental apoptosis activity is a physiological response in term pregnancy, it is observed that necrosis activity is increased in the placenta of preeclampsia and preterm birth. Necroptosis is a unique combination between necrosis and apoptosis, where necroptosis is actually a programmed cell death but has a necrosis‐like outcome, which leads to an inflammatory environment [5].

Necroptosis is thought to play a role in preeclampsia, which is also an inflammatory state. However, there have been very few studies evaluating necroptosis in preeclampsia. Necroptosis occurs through activation of a cascade involving *receptor-interacting serine/threonine-protein kinase* 1 (RIPK1), which then forms a complex with RIPK3 that leads to activation of *mixed lineage kinase domain-like pseudokinase* (MLKL) as an effector that increases cell permeability, thus cell lysis, which releases its content that triggers inflammation. RIPK1, RIPK3, and MLKL are therefore called the “necrosome” [6].

Maternal nutritional status is also associated with preeclampsia pathophysiology. One of the nutrients of interest is vitamin D, of which deficiency occurs in 99% of first trimester pregnant women in Jakarta, Indonesia [7]. In pregnancy, vitamin D in the form of 25‐hydroxycholecalciferol (25(OH)D) will also be converted to its active form 1,25‐dihydroxycholecalciferol (1,25(OH)_2_D3) in the placenta, meaning that the placenta actively metabolizes vitamin D to exert its function in the placenta and fetus. Vitamin D serves as a regulator, or rather a “fine tuner” of embryo implantation, immunosuppressed condition in early pregnancy, decidualization, and extravillous trophoblast (EVT) invasion. Vitamin D deficiency, defined as serum 25(OH)D less than 30 ng/mL, has been linked to pregnancy complications such as preeclampsia, IUGR, gestational diabetes mellitus, and preterm birth. To date, there has been no study that evaluates the association between the concentration of vitamin D and necroptosis activity in trophoblast.

## 2. Material and Methods

### 2.1. Study Populations

This was a cross‐sectional study to evaluate the association between placental vitamin D in the form of 25(OH)D and necroptosis activity in the placenta of normal patients and preeclamptic patients. The study was conducted during January 2021–May 2023. Subjects were enrolled in two hospitals in Jakarta, Indonesia: Cipto Mangunkusumo Hospital and Budi Kemuliaan Hospital. Subjects were divided into two groups, normal and preeclampsia. Inclusion criteria for the normal group were those with delivery at 37–41^+6^ weeks of gestational age (WGA), spontaneous, head presentation, lasting less than 18 h, without any maternal or fetal complication. Inclusion criteria for the preeclampsia group were those diagnosed with preeclampsia with severe features according to American College of Obstetrics and Gynecology 2020 guidelines. Subjects in the preeclampsia group received standard treatment of oral nifedipine as antihypertensive and magnesium sulfate 40%. Subjects with multiple pregnancies, other maternal comorbidities unrelated to preeclampsia, congenital anomalies, preterm premature rupture of membrane (PPROM), and incomplete data were excluded from the study. All patients agreed to written informed consent to participate in the study. This study has been approved by the ethical committee of the Faculty of Medicine, Universitas Indonesia–Cipto Mangunkusumo Hospital (Ethics Approval Number: ND.021/UN2.F1.DEPT.25/PDP.01/2023).

### 2.2. Samples

Samples taken for vitamin D analysis were 15 cc of venous blood and placenta from two marginal areas and two full thickness parenchymal areas for both vitamin D and necrosome analysis. Samples of blood and placenta were taken within 30 min following delivery. Blood samples were centrifuged and were kept in an −80°C freezer. For vitamin D analysis, one placental sample from each area was immersed in phosphate buffer saline (PBS), then was stored in −80°C. For necroptosis marker, one placental sample from each area was immersed in paraformaldehyde 4% solution, then embedded in paraffin blocks for examination. Analysis of serum and placental 25(OH)D was quantified by liquid chromatography‐tandem mass spectrometry (LC‐MS/MS) with Agilent liquid chromatography (LC) system 1290 in Prodia Laboratory, Jakarta. Necroptosis biomarkers, RIPK1, RIPK3, and MLKL, were performed in Bogor Institute of Agricultural. Placenta samples were made into paraffin blocks and were stained with hematoxylin eosin (HE) and immunohistochemistry (IHC) using anti‐RIPK1 antibody produced in rabbit for RIPK1, anti‐RIPK3 antibody produced in rabbit for RIPK3, and recombinant anti‐MLKL (phospho S358) antibody for MLKL. IHC blocks were analyzed using microscope Nikon type Eclipse 80i with 400× magnification on five fields of views of average staining in trophoblast cells and endothelium. Quantification was performed by a pathologist with blind system.

### 2.3. Analysis

Statistical analysis was performed using Statistical Package for the Social Sciences (SPSS) 23rd version. Quantitative variables were presented in mean and standard deviation if normally distributed, or median and minimum–maximum if abnormally distributed. Comparison between the two groups was performed using an unpaired *t*‐test if normally distributed, or Mann–Whitney test if abnormally distributed. Correlation test was performed using the Pearson test for normally distributed data, or the Spearman test if abnormally distributed. Qualitative test was performed using the chi‐square test for normally distributed data, or the Fisher test if abnormally distributed. Significant difference was considered if *p* < 0.05.

## 3. Results

### 3.1. Characteristics of Subjects

A total of 60 subjects participated in this study, of which 31 were assigned to the normal group, and 29 to the preeclampsia group. Characteristics of the subjects are summarized in Table 1. Gestational age upon delivery, birth weight, and placental weight were significantly different between groups. In the normal group, the median gestational age upon delivery was 38 weeks and 35 weeks in the preeclampsia group (*p* < 0.001). The mean birth weight in the normal group was significantly larger than that of the preeclampsia group (3080.33 ± 454.62 g vs. 2283.27 ± 833.63 g), as were the placentas (580.40 ± 129.36 g vs. 453.06 ± 173.65 g). There was no difference in body mass index or number of gravid.

**Table 1:** Characteristics of subjects.

	Normal (*n* = 31)	Preeclampsia (*n* = 29)	*p*
Maternal age (years)	30.73 (4.82)	31.51 (5.73)	0.482^a^
Gestational age (weeks)	38 (37–40)	35 (28–39)	< 0.001^b^
BMI (kg/m^2^)	24.15 (3.47)	25.81 (6.03)	0.174^a^
Gravid	2 (1–6)	2 (1–5)	0.730^b^
Parity	1 (0–4)	1 (0–4)	0.988^b^
Abortus	0 (0–1)	0 (0–2)	0.887^b^
Systolic pressure (mmHg)	110 (100–140)	180 (160–240)	< 0.001^b^
Diastolic pressure (mmHg)	70 (64–90)	110 (110–144)	< 0.001^b^
Birth weight (gram)	3080.33 (454.62)	2284.27 (833.63)	< 0.001^a^
Placental weight (gram)	580.40 (129.36)	453.06 (173.65)	< 0.001^a^

^a^paired *t*‐test.

^b^Mann–Whitney test.

### 3.2. Concentration of 25(OH)D and Placental Necrosome

Table 2 shows the concentration of serum and placental 25(OH)D, as well as placental necrosomes RIPK1, RIPK3, and MLKL. Placental 25(OH)D in the preeclampsia group was significantly lower than the normal group (15.00 [3.50–58.00] vs. 26.50 [5.00–153.00] ng/mL, *p* = 0.014), so was serum 25(OH)D, although not statistically significant. Based on the IOM classification, only six subjects (19.4%) in the normal group and two subjects (6.9%) in the preeclampsia group are with normal serum 25(OH)D concentration. A total of 93.1% of subjects in the preeclampsia group had vitamin D deficiency, compared with 80.6% in the normal group.

**Table 2:** Concentration of serum 25(OH)D and placental 25(OH)D, RIPK1, RIPK3, and MLKL in the normal and preeclampsia groups.

	Normal (*n* = 31)	Preeclampsia (*n* = 29)	*p*
Serum 25(OH)D (ng/mL)	21.03 (10.06)	16.49 (9.67)	0.138^a^
Normal (> 30 ng/mL)	6 (19.4%)	2 (6.9%)	0.156^c^
Deficiency	25 (80.6%)	27 (93.1%)	
Placental 25(OH)D (ng/g)	26.50 (5.00–153.00)	15.00 (3.50–58.00)	0.014^b^
Placental RIPK1			
Trophoblast	31.15 (7.70–103.20)	19.60 (5.10–109.50)	0.108^b^
Endothelium	4.00 (0.20–24.80)	2.60 (0–23.60)	0.169^b^
Placental RIPK3			
Trophoblast	76.20 (20.59)	93.88 (23.94)	0.003^a^
Endothelium	30.93 (18.33)	39.13 (18.66)	0.086^a^
Placental MLKL			
Trophoblast	4.40 (1.30–19.10)	4.10 (1.30–41.20)	0.819^b^
Endothelium	0.90 (0.00–8.80)	0.80 (0–12.00)	0.721^b^

^a^paired *t*‐test.

^b^Mann–Whitney test.

^c^Chi‐square test.

**Figure 1:**
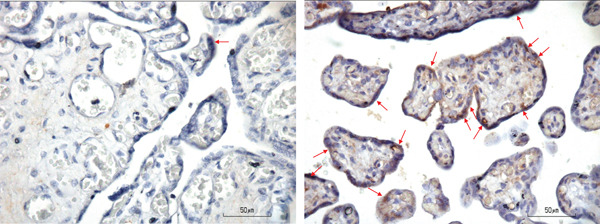
RIPK1 staining of the normal group (left, arrow) and the preeclampsia group (right, arrow).

**Figure 2:**
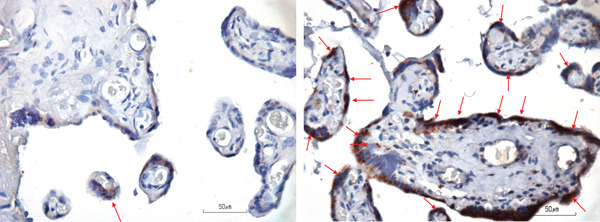
RIPK3 staining of the normal group (left, arrow) and the preeclampsia group (right, arrow).

**Figure 3:**
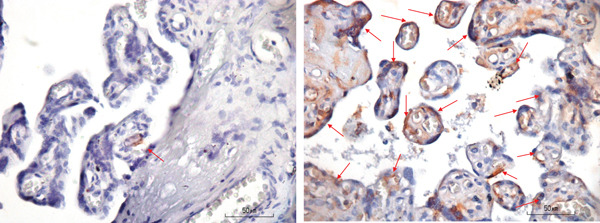
MLKL staining of the normal group (left, arrow) and the preeclampsia group (right, arrow).

Placental necrosome was examined at trophoblast and endothelial. IHC samples of placenta for RIPK1, RIPK3, and MLKL were shown in Figures 1, 2, and 3, respectively. Only trophoblast RIPK3 was found to be significantly higher in the preeclampsia group compared with the normal group (93.88 [23.94] vs. 76.20 [20.59], *p* = 0.003), whereas endothelial RIPK3 was also higher in the preeclampsia group albeit with no statistical significance.

### 3.3. Correlation Between 25(OH)D and Necrosomes

Correlation between serum and placental 25(OH)D with placental necrosomes RIPK1, RIPK3, and MLKL is shown in Table 3. A positive correlation was found between serum and placental 25(OH)D (*r* 0.253, *p* = 0.026). Placental 25(OH)D was found to have a negative correlation with trophoblast RIPK3 (−0.352, *p* = 0.003), endothelial RIPK3 (*r* = −0.244, *p* = 0.03), and trophoblast MLKL (*r* = −0.296, *r* = −0.011). There was no correlation between serum 25(OH)D and any necrosome.

**Table 3:** Correlation of serum 25(OH)D with placental necroptosis biomarkers.

Variables	*r*	*p*
Serum 25(OH)D–placental 25(OH)D	0.253	0.026^a^
Serum 25(OH)D–		
Trophoblast RIPK1	0.171	0.096^a^
Endothelial RIPK1	0.100	0.224^a^
Trophoblast RIPK3	0.156	0.117^a^
Endothelial RIPK3	0.212	0.052^a^
Trophoblast MLKL	0.048	0.359^a^
Endothelial MLKL	−0.028	0.416^a^
25(OH)D placenta–		
Trophoblast RIPK1	0.079	0.275^b^
Endothelial RIPK1	−0.043	0.371^b^
Trophoblast RIPK3	−0.352	0.003^a^
Endothelial RIPK3	−0.244	0.030^a^
Trophoblast MLKL	−0.296	0.011^b^
Endothelial MLKL	−0.112	0.196^b^

^a^Pearson′s correlation test.

^b^Spearman′s correlation test.

## 4. Discussion

Preeclampsia is a pregnancy‐specific hypertensive condition involving a multiorgan system, associated with increased both maternal and fetal mortality and morbidity. In this study, the preeclampsia group had lower means of gestational age. The risk of preterm birth is over four times greater in preeclampsia, mostly due to iatrogenic cause. However, spontaneous preterm labor is also observed in preeclampsia, due to increased oxidative stressed and inflammation [8]. Lower birth weight was also observed in the preeclampsia group. Besides higher preterm delivery, lower birth weight is also attributable to impaired fetal perfusion thus inadequate nutrition delivery, leading to fetal growth restriction [9]. Lower placental weight in the preeclampsia group is in accordance with the finding of Akhlaq et al., where compared with normal placenta, preeclamptic placenta is macroscopically lighter, smaller, and thinner; microscopic findings include increased necrosis, infarct, syncytial knot, hypertrophy of the spiral artery smooth muscle, and hypoplasia of the distal villi. However, lower placental weight observed in preeclampsia group can also be due to prematurity [5]. Placental 25(OH)D concentration in the preeclampsia group was significantly lower compared with the normal group. Serum 25(OH)D in the preeclampsia group was also lower, although not statistically significant. The mild positive correlation of maternal serum and placental 25(OH)D might reflect that besides maternal vitamin status, vitamin D is actively metabolized in it. It metabolizes vitamin D with the presence of vitamin D receptor (VDR) and CYPB21 enzyme converting 25(OH)D into its active form 1,25(OH)2D3. The role of vitamin D in pregnancy starts from implantation phase, regulating HOXA10 gene that is associated with endometrial receptivity and decidualization, regulating immune response particularly the activity of natural killer (NK) cells, dendritic cells, macrophage, and T cells. The metabolism of vitamin D in EVT supports trophoblast invasion, whereas in syncytiotrophoblast, vitamin D regulates the production of human chorionic gonadotropin (hCG), human placental lactogen (hPL), and progesterone, creating an anti‐inflammatory environment [10]. Physiologically, as gestation advances, placental vitamin D metabolism shifts from local immunomodulation towards fetal supply and labor preparedness, characterized by reduced activation, increased degradation, and increased transfer of vitamin D to the fetus. In our study, serum 25(OH)D was not significantly lower in the preeclampsia group, which is in accordance with several studies. Besides the low maternal serum of vitamin D, it is thought that the low placental concentration of vitamin D is attributable to dysregulation of VDBP endocytosis and vitamin D metabolism [11, 12]. In preeclampsia, there is an increased rate of 25(OH)D3 catabolism into its inactive form 24,25(OH)_2_D3 through expression of 24‐hydroxylase enzyme. Moreover, active metabolite form of vitamin D in preeclampsia is dominantly 3‐epi‐1,25(OH)_2_D3 instead of 1,25(OH)_2_D3, which shows weaker affinity to VDR [12]. According to the IOM criteria, both groups had a mean of maternal serum of vitamin D in deficiency category (normal vs. preeclampsia, 21.03 [10.06] vs. 16.49 [9.67] ng/mL), which were lower than the normal minimum value of 30 ng/mL [13, 14]. Vitamin D deficiency is associated with increased risk of preeclampsia through several mechanisms: increased synthesis and response towards proinflammatory cytokines, impaired energy metabolism in mitochondria, increased rate of cell deaths, impaired regulation of gene transcription–translation associated with implantation and EVT, disruption of angiogenesis as well as its structure and elasticity of the vessels, imbalance of renin–angiotensin–aldosterone axis thus unregulated blood pressure, and increased reactive oxygen species (ROS). The adequacy of serum concentration of vitamin D has been associated with decreased risk of preeclampsia for up to 40% [15–19]. In addition to normal serum levels of vitamin D having lower risk of preeclampsia, Mirzakhani et al. also reported that 348 vitamin D‐associated genes were differentially expressed (158 of which upregulated) in peripheral blood of preeclampsia patients. These vitamin D‐associated genes suggested several highly functional modules associated with systematic inflammatory responses [20]. Placental necrosome RIPK3 in the preeclampsia group was higher compared with the normal group (93.88 [23.94] vs. 76.20 [20.59], *p* = 0.003) in trophoblast and endothelial, although the latter did not have a significant difference [21]. Increased necroptosis activity has been found in several diseases in various organ system, among which are cancer, renal fibrosis, rheumatoid arthritis, psoriasis, inflammatory bowel disease (IBD), nonalcoholic fatty liver disease, myocardial infarction, asthma, and chronic obstructive pulmonary disease [22]. The diseases known to have increased necroptosis activity are typically high inflammatory, which is also a character of preeclampsia [6]. This study was in accordance with Bailey et al. who found an increased necroptosis activity in both early onset and late onset preeclampsia, marked by increased RIPK1, RIPK3, and MLKL. RIPK3 was found higher in early onset preeclampsia. Increased necroptosis in preeclampsia is thought to be associated with the caspase 8 deficiency, which is crucial for cytotrophoblast fusion into syncytiotrophoblast. In addition, caspase 8 is also needed for a cell to undergo apoptosis, where its deficiency will divert the pathway into necroptosis, leading to excessive oxidative stress [23, 24]. Necroptosis cascade can be started through the activation of RIPK1 or directly through RIPK3. TNF receptor 1 (TNF‐R1), CD95, and DR4/5 are RIPK1‐specific precursors, whereas TLR3/4 and ZBP‐1 are RIPK3‐specific [20]. Based on this theory, it is hypothesized that necroptosis in preeclampsia is a RIPK3‐dependent one, which does not need RIPK1 activation [25, 26]. Besides necroptosis, RIPK1 also plays a role in apoptosis in the presence of caspase 8. Our study did not find an increased RIPK1 in the preeclampsia group, which might suggest that apoptosis is not a dominant pathway of cell death in preeclampsia [22]. RIPK3 has been linked with increased inflammasomes, which creates an inflammatory environment, which is the character of preeclampsia [27]. In contrast with Bailey et al., our study did not find different MLKL concentration between the two groups. This study was designed to compare normal pregnancy with median gestational age 38 weeks and preeclampsia with median gestational age 35 weeks. This finding might suggest that necroptosis, just like apoptosis, is indeed a physiological phenomenon, which occurs excessively or prematurely in preeclampsia. The aim of these various cell death mechanisms is maintaining organ homeostasis to preserve “good” cells and discard “impaired” cells. Indeed, deletion of RIPK1 gene was found to be lethal, according to Degterev et al. Any aberrancy of these processes will lead to pathological condition, such as preeclampsia [28–31]. Placenta is an organ that actively metabolizes placenta, as shown by the positive correlation between serum and placental 25(OH)D in our study (*r* 0.253, *p* = 0.026). Fetus cannot produce its own 25(OH)D, so it relies on circulating 25(OH)D in maternal serum that goes to syncytiotrophoblast through controlled endocytosis with membrane plasma megalin/cubilin. Most of 25(OH)D will be metabolized in placenta, leaving small portion goes into fetal circulation. Syncytiotrophoblast has CYP27B1 enzyme that metabolizes 25(OH)D into active form 1,25(OH)_2_D_3_, which facilitates gene transcription or be transferred to fetal or maternal circulation [32].Our study showed mild‐to‐moderate negative correlation between placental 25(OH)D with trophoblast RIPK3 (−0.352, *p* = 0.003), endothelial RIPK3 (*r* = −0.244, *p* = 0.03), and trophoblast MLKL (*r* = −0.296, *r* − 0.011). These findings suggest that lower placental 25(OH)D is associated with increased trophoblast necroptosis activity. To date, there has been no study about the role of vitamin D towards necroptosis activity in preeclampsia. However, there have been several studies linking vitamin D and necroptosis in other pathology such as IBD and tuberculosis, which also confirmed the protective effect of vitamin D through decreasing RIPK3 concentration [21, 33]. Vitamin D has several roles in regulating necroptosis. In RIPK1‐dependent necroptosis, VDR that has been coupled with vitamin D will bind with RIPK1, which prevents further cascade activation [21]. In RIPK3‐dependent necroptosis, vitamin D interacts with ligase E3 as a basal threshold regulator of RIPK3 to commence the cascade [34]. Vitamin D is also reported to produce caspase 3, which shifts cell death tendency towards apoptosis, therefore reducing necroptosis [33]. The evidence of vitamin D in maintaining healthy pregnancy has been abundant, one of which is the increased vitamin D production and consumption in vivo starting as soon as placental implantation occurs. These are the rationale of supplementing vitamin D as early as possible, with maximum protective effects in the preconception period. Dosing recommendation is beyond the scope of this research; however, Hollis et al. reported that 4400 IU/day of vitamin D is demonstrated to be safe, able to maintain circulating level of serum 25(OH)D of 40 ng/mL and without any adverse event reported [34].This study has several limitations. Firstly, the cross‐sectional design by its nature cannot evaluate causality between observed variables. Secondly, this study only measured 25(OH)D as a representative of vitamin D status, which was not the active metabolite 1,25(OH)_2_D_3_, and did not measure the VDR and enzyme CYP27B1 or CYP24A1 that might reflect more about its metabolism. Lastly, the IHC staining was examined by visual evaluation, in which subjective bias cannot be ignored, although it was done with blinding method.

## 5. Conclusion

To conclude, this study found that compared with the normal group, the preeclampsia group had a lower concentration of placental 25(OH)D and a higher concentration of necrosome RIPK3. This study also found a mild‐to‐moderate negative correlation between 25(OH)D and trophoblast RIPK3, endothelial RIPK3, and trophoblast MLKL, suggesting an association between lower placental vitamin D levels and increased trophoblast necroptosis activity. Further research about the role of vitamin D in regulating necroptosis in preeclampsia is warranted.

## Author Contributions

Noroyono Wibowo and Rima Irwinda contributed in conceptualization, data collection, funding acquisition, and providing revisions. Atikah Sayogo Putri contributed in data collection, data analysis, and interpretation.

## Funding

No funding was received for this manuscript.

## Conflicts of Interest

The authors declare no conflicts of interest.

## Data Availability

The data that support the findings of this study are available on request from the corresponding author, A.S.P. The data are not publicly available due to their containing information that could compromise the privacy of research participants.
